# Parents’ Sport Socialization Values, Perceived Motivational Climate and Adolescents’ Antisocial Behaviors

**DOI:** 10.5964/ejop.v15i4.1598

**Published:** 2019-12-20

**Authors:** Francesca Danioni, Daniela Barni

**Affiliations:** a Family Studies and Research University Centre, Catholic University of Milan, Milano, Italy; b Department of Human Sciences, LUMSA University of Rome, Rome, Italy; University of Liverpool, Liverpool, United Kingdom

**Keywords:** adolescents’ antisocial behaviors, parents’ sport values, perceived motivational climate, relative importance, team sport

## Abstract

Parents play a key role in young athletes’ sport experience. In particular, parents’ sport goals for children may influence young athletes’ morally relevant sport behaviors. The present study involves 172 Italian adolescents (female = 51.7%; age M = 15.41, SD = 1.73) practicing team sports and analyzed whether and the extent to which parents’ sport socialization values, those values adolescents perceived their parents wanted them to endorse (i.e., moral, competence, status values), were associated with young athletes’ antisocial behaviors towards teammates and opponents. Adolescents’ perceptions of the prominent motivational climate (i.e., mastery and performance) within their team were also considered. Participants were asked to fill out questionnaires, including the Youth Sport Values Questionnaire-2, adapted to measure adolescents’ perceptions of parental socialization values, the Perceived Motivational Climate in Sport Questionnaire and the Prosocial and Antisocial Behavior in Sport Scale. The results of multiple linear regression analysis and relative weight analysis showed that mastery motivational climate, as protective factor, and mothers’ status values, as risk factor, were the most important variables in predicting adolescents’ antisocial behavior towards teammates. As far as adolescents’ antisocial behavior towards opponents was concerned, performance motivational climate and mothers’ status values were the most relevant predictors: the more adolescents perceived their coaches and mothers as giving importance to performance and status, the higher was the frequency of their antisocial behavior in sport. Implications and further developments of the study are discussed.

The extent of morally-relevant topics in youth sport is a question of considerable cultural, psychological and educational interest (Shields, Bredemeier, LaVoi, & Power, 2005). In recent years, psychosocial research has devoted growing attention to the role of sport in the development not only of young athletes’ physical abilities, but also of moral attitudes and values. Indeed, it has been widely proposed that sport develops character (Lee, Whitehead, Ntoumanis, & Hatzigeorgiadis, 2008; Shields & Bredemeier, 1995).

Competitive sports are often addressed as a potentially positive environment for youth (Larson, 2000), since they create opportunities to teach young athletes beneficial mental skills (e.g., Steiner, McQuivey, Pavelski, Pitts, & Kraemer, 2000) and to transmit relevant human values (e.g., Danioni, Barni, & Rosnati, 2017). However, competitive sports may also be a negative environment for youth by promoting undesirable behaviors (e.g., Kavussanu, Seal, & Phillips, 2006). Due to their social nature, sport contexts may provide occasions for prosocial but also for antisocial behaviors, such as cheating, or injuring an opponent on purpose, or verbally abusing a teammate (Kavussanu, 2008).

Given the various possible outcomes of team sport participation during youth and the active role sport holds in the socialization process (Kremer-Sadlik & Kim, 2007), the present study aimed at analyzing the relation between parents’ socialization sport values (i.e., the values related to sport that parents would like their children to endorse), the team’s motivational climate, and young athletes’ morally relevant behaviors (specifically, antisocial behaviors towards teammates and opponents). This will be done by considering a group of Italian adolescents continuously practicing a team sport.

## Parents’ Role in Children’s Sport: The Socialization Values

Human values, which influence individual decision making on a day-to-day basis, have been defined as “trans-situational goals that vary in importance and serve as guiding principles in the life of a person or a group” (Schwartz, 2007, p. 712). Because they are trans-situational, they influence attitudes and behaviors in the many contexts—including sports—of an individual’s life (Lee et al., 2008). However, only in recent years has psychosocial research focused on the internalization of values through sports, and on behaviors in sport contexts by young athletes (e.g., Danioni et al., 2017; Danioni & Barni, 2017; Holt, Tink, Mandigo, & Fox, 2008).

Studies dealing with young athletes’ personal values show consistent results. Young athletes tend to give more importance to competence (e.g., achievement and skill) and moral values (e.g., contract maintenance and obedience) than to status values (e.g., public image and winning) (e.g., Danioni et al., 2017; Goggins, 2015; Lee et al., 2008). These values were found to be related to athletes’ prosociality and antisociality. Athletes’ status values support antisocial behavior and attitude, whereas moral values promote prosociality (Lee et al., 2008; Lucidi et al., 2017). Some of the studies considered were carried out in the Italian context, which is the specific focus of our research. Similarly, Šukys and Jansonienė (2012), in their study involving 318 university students practicing sport at national or international level, found that moral sport values are negatively correlated to moral disengagement in sport.

Compared to athletes’ personal values, the topic of parents’ values in shaping their children’s sport experience has received less attention so far, this despite the fact that the available literature shows that parents play a key role in children’s sport activity (e.g., D’Arripe-Longueville, Pantaléon, & Smith, 2006). It has been recognized that young athletes are greatly influenced by parental attitudes towards their child’s sport performance (Gould, Lauer, Rolo, Jannes, & Pennisi, 2008; Kavussanu, White, Jowett, & England, 2011). Parental influence on children’s sport activity has been addressed mainly in terms of parental involvement (e.g., Teques, Serpa, Rosado, Silva, & Calmeiro, 2018). Hellstedt (1987) theorized parental involvement as a continuum ranging from underinvolvement to overinvolvement. Both ends of the continuum may have negative effects on adolescent players: in case of underinvolvement, adolescents can feel a lack of emotional, financial or functional investment on the part of their parents, thus making it more difficult for them to pursue a career in sport. In case of overinvolvement, instead, parental pressure can be harmful, as the parents’ needs are satisfied through their children’s sport activity and parents may not be able to differentiate their own expectations from those of their children. Of course, the quality of the parent-child relationship influences an adolescent’s perceptions of his/her parent’s attitude towards the sport activity practiced (Lee & MacLean, 1997). Parental praise and understanding towards children’s sport activity (Lee & MacLean, 1997), which elicit children’s perception of parental empathy displayed towards the sport they practice, promote an increase in players’ enjoyment and motivation for sport (Sánchez-Miguel, Leo, Sánchez-Oliva, Amado, & García-Calvo, 2013), as well as facilitate the transmission of values (Danioni et al., 2017). In contrast, parental pressure in the sport context might enhance feelings of distress, guilt and burnout (Donnelly, 1993; Udry, Gould, Bridges, & Tuffey, 1997), and cause a decrease of enjoyment (Anderson, Funk, Elliot, & Smith, 2003).

Also parental goals and beliefs about their children’s sport experience have been investigated, even if to a lesser extent: Athletes’ perception of parental beliefs regarding effort, learning, and enjoyment versus outcome indirectly influences athletes’ achievement-related cognitions and personal beliefs (White, Kavussanu, Tank, & Wingate, 2004). Goggins (2015) emphasized instead the role of parents’ values in shaping their children value priorities: Indeed, parents’ own values and children’s perceptions of their parents’ values significantly predict a child’s own status values, namely those values that prioritize public image and winning.

Goggins (2015) has focused on the influence of parents’ own sport values and on children’s perceptions of their parental sport values, but, to our knowledge, no studies have focused on children’s perceptions of their parents’ sport socialization values. Parental socialization sport values as perceived by their children can be defined as the values children believe their parents want them to endorse with regard to the sport activity they practice. Indeed, children may consider their parents as wanting them to endorse specific sport values and this might have an impact on the way they experience and behave within the sport context. Generally speaking, parental socialization values may be different from parents’ personal values (Barni, Rosnati, & Ranieri, 2013; Tam, Lee, Kim, Li, & Chao, 2012), especially when parents recognize that their children are growing up in a social context that differs from the one in which they themselves were reared (Alwin, 1988) and that they need to prepare their children for social life (e.g., Benish-Weisman, Levy, & Knafo, 2013). Parents may therefore modify their socialization values from their personal values to make them correspond to what they think is really beneficial for their children (Knafo & Galanski, 2008).

## Coaches' Role in Children’s Sport: The Perceived Motivational Climate

Coaches enhance young athletes’ strengths and personal resources (Côté & Gilbert, 2009; Fraser-Thomas, Côté, & Deakin, 2005), and their conduct during trainings and competitions influences young athletes’ motivation to participate (Rottensteiner, Laakso, Pihlaja, & Konttinen, 2013; Smith, Quested, Appleton, & Duda, 2017). However, coaches might also induce anxiety and burnout and their actions may drive adolescents to drop out from sport (Smith, Smoll, & Cumming, 2007; Weiss, Amorose, & Wilko, 2009). Interestingly, the coaches’ influence has been attributed, at least in part, to the motivational climate they create (Jõesaar, Hein, & Hagger, 2012). Perceived motivational climate in achievement contexts tends to activate self- versus other-referenced criteria for the evaluation of competence (Nicholls, 1989). The first attempts to investigate the motivational climate derive from the educational context. Originating from the school domain (Ames & Archer, 1988), the situational goal structure, namely the motivational climate, has been distinguished into mastery climate, where the emphasis of the context is on participation, individual progress and task mastery, and performance climate, where the emphasis is instead on normative success and outperforming others. More recently, in the sport context the perception of motivational climate within the team has been found to deeply influence young athletes’ sport experience (e.g., Fry & Gano-Overway, 2010), for example with regard to the desire to continue playing. Boiché and Sarrazin (2009) found the mastery-oriented team motivational climate to be positively associated with sport persistence. On the contrary, athletes’ perceptions of a performance-oriented motivational climate were associated with dropout.

## How do Parents and Coaches Influence Children’s Moral Behaviors in Sport?

To our knowledge parental influence has been scarcely investigated in relation to children’s moral behaviors in sport. One of the few exceptions is the work carried out by Guivernau and Duda (2002), who argued that athletes’ perceptions of their parents’ approval for cheating and aggression (e.g., trying to injure an opponent) is related to athletes’ views about appropriate behavior within sports. According to the authors, parents—together with the coaches—“shape” the moral atmosphere operating in youth sport teams. Stuart and Ebbeck’s study (1995), involving athletes aged 9–15, found instead that for adolescents (grades 7 and 8), the perceptions of significant others’ (including the parents) approval for antisocial behavior are inversely related to reason, prosocial behavior and the intent to exhibit moral behavior. With regard specifically to morally relevant behaviors, parental goals that promote a performance-oriented climate were found to discourage participants’ prosocial sport behavior and to encourage acceptance of cheating (Wagnsson, Stenling, Gustafsson, & Augustsson, 2016), and a father-initiated performance climate was found to be positively associated with athletes’ antisocial sport behaviors (LaVoi & Stellino, 2008).

Adolescents’ perceptions of their team’s goals, promoted mainly by the coach, also need to be taken into consideration when analyzing the multiple sources of adolescents’ moral behaviors (e.g., Kavussanu, Roberts, & Ntoumanis, 2002; Miller, Roberts, & Ommundsen, 2005; Nicholls, 1989; Rutten et al., 2011; Shields & Bredemeier, 1995). Indeed, Nucci and Kim (2005) identified the coach as an important person within the sport context, who is also in a position to influence adolescents’ social behavior. Not only are young athletes’ personal goal orientations assumed to mediate the effects of values on their prosocial and antisocial attitudes and cheating (Lee et al., 2008; Lucidi et al., 2017), but also the motivational climate promoted by the coaches is likely to influence morally relevant behaviors in youth sport.

Recent works on this topic have shown that the perception of a mastery climate is positively related to sportspersonship orientations (D’Arripe-Longueville et al., 2006; Wells, Ellis, Arthur-Banning, & Roark, 2006), such as commitment, respect for social conventions, for rules and officials (Miller et al., 2005). In a sample of 279 soccer players aged 12–14 years, perceptions of a mastery climate were found to be positively associated with moral reasoning, and negatively with amoral intentions and behaviors (Ommundsen, Roberts, Lemyre, & Treasure, 2003). In a performance climate, instead, athletes are more likely to engage in inappropriate behaviors (Bortoli, Messina, Zorba, & Robazza, 2012).

To our knowledge, only a few studies have investigated how parents’ and coaches’ goals interact to affect moral behavior in young athletes (e.g., Guivernau & Duda, 2002; Stuart & Ebbeck, 1995). However, this is an important topic since young athletes’ moral development is exposed to both parents’ and coaches’ desirable or undesirable influences. Moreover, in order to address the topic of morally relevant behaviors in sport in terms of antisocial behavior, it is important to distinguish with regard to the recipient of the specific behavior (teammates vs. opponents) (Kavussanu & Boardley, 2009). In particular, it is likely that teammates being an ideal example of an in-group elicit more positive behaviors compared to opponents (the out-group) (Danioni & Barni, 2017).

## Present Study

In the light of all the above considerations, the present study mainly aims at understanding the role of parental socialization values with regard to sport, as perceived by adolescent children, in shaping their antisocial behaviors towards teammates and opponents. As already mentioned, parents’ sport socialization values refer to those sport values (i.e., moral, competence, status) adolescents perceive their parents want them to endorse. The review of the literature clearly suggested that, in order to consider the different sources of antisocial behavior in youth sport, we cannot exclude adolescents’ perceptions of the goal structures created by their coach, namely the perceived motivational climate of their team (Kavussanu, 2006). Based on the goals young athletes perceive as being promoted within their team, the sport motivational climate has been distinguished into mastery climate, where the emphasis of the context is on participation, individual progress and task mastery, and performance climate, where the emphasis is instead on normative success and outperforming others. We therefore considered both parental socialization values with regard to sport and motivational climate in shaping young athletes’ antisocial behavior. By using a relatively recent analytical strategy, that is, the Relative Weight Analysis (RWA) (Johnson, 2000), which is able to take the interrelations among variables into account, we addressed three main questions: “How much of the variation among adolescents with regard to antisocial behaviors can be explained by parents’ sport socialization values and by the team’s motivational climate?”; “Which is the most important predictor of adolescents’ antisocial behaviors: The parents’ sport socialization values or the team’s motivational climate?”; “Are there differences in predicting antisocial behavior in sport when the recipient is a teammate versus an opponent?”

For the first question, we expected the variables under examination to make a large contribution to explain adolescents’ antisocial behaviors towards both teammates and opponents. In particular, adolescents’ perceptions that their parents attribute importance to moral values should discourage the adoption of antisocial behaviors (H1). In contrast, we expected that the more adolescents perceived their parents as wanting for them to win and to promote their public image (status values) the more they would adopt antisocial behaviors in sport contexts (H2). These two hypotheses are in line with the available literature dealing with adolescents’ personal values (e.g., Danioni & Barni, 2017; Lee et al., 2008), since to our knowledge, no information is available on parental sport socialization values. Based on the available literature on team motivational climate (e.g., Kavussanu, 2006; Sage & Kavussanu, 2008), we expected performance climate to be positively related to antisocial behaviors (H3) whereas athlete’s perception of a mastery climate was expected to be negatively linked to antisocial behaviors (H4).

In relation to the second question, despite the coach and the motivational climate promoted tend to emerge as important sources in influencing young players’ decisions to engage in an antisocial behavior (Guivernau & Duda, 2002; Nucci & Kim, 2005), we can hypothesize the significant role played also by parents in influencing these behaviors (e.g., Danioni & Barni, 2017). We expect therefore both to be related to this specific behaviors (H5).

With regard to our third question we expected motivational climate (both mastery and performance oriented—although with different patterns) to have greater implications for antisocial behaviors towards teammates rather than opponents (H6), as a within-team variable is more likely to influence within-team behaviors rather than between-teams behaviors (e.g., Boardley & Kavussanu, 2009).

## Method

### Participants and Procedure

Participants were 172 adolescents (51.7% female; age 13–19, *M* = 15.41, *SD* = 1.73), from northern and central Italy, continuously participating in team sports. Most of the participants were born in Italy (97%), while 3% was born abroad (Albania, Ethiopia, France, Philippines). Eighty-nine percent of the adolescents lived with both parents, while the remaining 11% lived with only one parent (in general, the mother). Most participants (86.6%) had at least one sibling (*M* = 1.31, *SD* = 0.62).

Their mean age at the beginning of their sport activity was 6.20 years (*SD* = 2.17). The majority of the participants played volleyball (60.4%), followed by soccer (19.8%), basketball (12.2%) and rugby (7.6%). All adolescents were amateur athletes.

Participants were recruited with the collaboration of their sport teams and were informed by letter about the main objectives of the present study. Those adolescents whose parents consented individually filled out a self-report questionnaire either before or after a regular training session, in the presence of both of their coach and a research team member (response rate: 86%). In case of questions or concerns, they were addressed to the research staff member and not to the coach, who was not allowed to see participants’ responses. Participants were told that their responses were completely anonymous. The study was approved by the Scientific Committee of the Family Studies and Research University Centre, Catholic University of Milan, Italy, and followed the APA ethical guidelines for research.

### Measures

#### Socio-Demographic Information

Participants were asked to provide personal information (age, gender, and country of birth) and family characteristics (family structure and number of siblings), as well as details of their sport activity (age they began the sport activity and type of sport practiced).

#### Adolescents’ Perceptions of Parents’ Sport Values

The Youth Sport Values Questionnaire-2 (YSVQ-2; Lee et al., 2008) was adapted to measure adolescents’ perceptions of those sport values their parents wanted them to endorse. The original 13-item instrument intends to assess the importance athletes give to three core sport value dimensions (moral, competence and status values). In this study, adolescents were asked to rate how much their own parents wanted them to give importance to each value (e.g., “How important is it for your father/mother that you try to be fair?”) on a 7-point Likert scale (from −1 = the opposite of what my father/my mother would like to 5 = extremely important to my father/my mother). Adolescents answered twice, once for fathers and once for mothers. The importance score for each value dimension was calculated as the mean of the items tapping that perceived parents’ sport value dimension: moral values (α = .85 for fathers and α = .88 for mothers), competence values (α = .74 for fathers and α = .80 for mothers) and status values (α = .83 for fathers and α = .81 for mothers). The items used were the ones translated into Italian; the original version of the scale, aimed at measuring one’s own personal values, supported the three factors structure of the scale that showed satisfactory internal consistency (Lee et al., 2008).

#### Perceived Motivational Climate

The Perceived Motivational Climate in Sport Questionnaire–12 (PMCSQ–12; drawn from Newton, Duda, & Yin, 2000; for the Italian version of the scale, see Bortoli & Robazza, 2004) was used to measure adolescents’ perception of the prominent motivational climate. The 12-item scale aims at assessing two kinds of sport motivational climates, namely mastery (e.g., “On this team, each player contributes in some important way”) and performance climate (e.g., “On this team, only the top players ‘get noticed’ by the coach”). Participants were asked to rate on a 5-point scale (from 1 = strongly disagree to 5 = strongly agree) the extent to which they perceived the climate described within their sport team. Both dimensions showed acceptable internal consistency (α = .74 for mastery climate, α = .71 for performance climate). The two-factor structure of the Italian version of the scale was supported in a study carried out on almost 600 soccer players aged between 16 and 25 and showed satisfactory reliability (Bortoli & Robazza, 2004).

#### Adolescents’ Antisocial Behavior

An Italian translation of the subscales of antisocial behavior towards teammates and opponents drawn from the Prosocial and Antisocial Behavior in Sport Scale (PABSS) (Kavussanu & Boardley, 2009) was used. Adolescents were asked to rate the frequency with which they adopted each behavior described on a 5-point Likert scale (from 1 = never to 5 = very often). Examples of items are “While playing sport this season, I verbally abused a teammate” (antisocial behavior towards a teammate) or “While playing sport this season, I intentionally distracted an opponent” (antisocial behavior towards an opponent). Both dimensions showed acceptable internal consistency (α = .79 for antisocial behavior towards teammates, α = .78 for antisocial behavior towards opponents). This is a widely recognized measure aimed at assessing this construct, which showed good convergent, concurrent, and discriminant validity together with good test–retest reliability (Kavussanu, Stanger, & Boardley, 2013).

#### Data analysis

##### Preliminary Analysis

The study variables were described in terms of means, ranges and standard deviations; bivariate Pearson correlations between them were calculated.

##### Predicting Antisocial Behaviors: Adolescents’ Perceptions of Parents’ Sport Values and Perceived Motivational Climate

To assess whether and the extent to which parental sport values and the motivational climate, as perceived by adolescents, were associated with adolescents’ antisocial behaviors towards teammates and opponents, both multiple regression (MR) and Relative Weight Analysis (RWA) were performed, with the three parental sport socialization values and the two dimensions of perceived motivational climate as predictors, separately for antisocial behavior towards teammates and opponents as criterion variables.

The MR was used in order to estimate the overall *R*^2^ and determined the statistical significance of individual regression coefficients. Regression coefficients inform as to the extent to which the criterion variable would change based on a given increase in a predictor while the other predictors are held constant (i.e., unique contribution). However, when predictors are highly correlated—as is likely in the case of the parental sport values and of the motivational climate—MR is not enough to adequately divide variance in the criterion among the predictors (Kraha, Turner, Nimon, Reichwein Zientek, & Henson, 2012). When faced with correlated predictors, researchers should therefore combine MR with other techniques available for interpretation, such as RWA (Johnson, 2000). RWA focuses on the impact of a particular predictor relative to others in the model: that is, the proportionate contribution each predictor makes to *R*^2^, taking into account both the unique relationship with the criterion and its relationship when combined with other predictors (i.e., relative contribution). Relative weights can be estimated by creating a set of variables that are highly related to the original one but are uncorrelated with each other. The criterion variable can then be regressed on the new uncorrelated variables to approximate the relative weights of the original variables (for further details, see Barni, 2015; Johnson, 2000). The importance weights provided by the analysis can then be scaled in the metric of relative effect size by dividing the relative weights by the model *R*^2^ and then multiplying these values by 100. In this way, the rescaled weights are interpreted as the percentage of predicted criterion variance attributed to each predictor.

## Results

### Preliminary Analysis

Means and standard deviations of the study variables are reported in Table 1.

**Table 1:** Adolescents’ Perceptions of Their Parents’ Sport Values, Mastery and Performance Climate, and Antisocial Behaviors Towards Teammates and Opponents

Variable	Moral Values		Competence Values		Status Values		Mastery Climate	Performance Climate	Antisocial Behaviors	
	FA	MO	FA	MO	FA	MO			Towards Teammates	Towards Opponents
*M*	3.66	3.58	3.51	3.26	1.75	1.45	4.12	2.60	2.12	2.27
Range	.0 to 5.0	−1.0 to 5.0	.0 to 5.0	−1.0 to 5.0	−.8 to 5.0	−1.0 to 5.0	1.67 to 5.0	1.00 to 4.33	1.00 to 4.60	1.00 to 4.83
*SD*	1.07	1.23	1.06	1.21	1.51	1.39	0.60	0.76	0.78	0.87

Note: FA = Fathers; MO = Mothers.

Generally speaking, adolescents perceived their fathers and mothers to give great importance to moral values and to competence values, and little importance to status values. They also perceived their team to be highly mastery oriented, rather than characterized by a performance oriented motivational climate. Finally, adolescents reported low to moderate levels of antisocial behaviors.

In Table 2 we reported the correlations between the study variables. The correlation coefficients range from −.42 (*p* < .01) between mastery and performance motivational climate to .79 between mothers’ and fathers’ sport status values (*p* < .01).

**Table 2:** Person Bivariate Correlations Between the Study Variables

Variable	1	2	3	4	5	6	7	8	9	10
1. Mother’s moral values	-	.62**	.08	.61**	.33**	−.11	.32**	−.15	−.31**	−.17*
2. Mother’s competence values		-	.49**	.39**	.69**	.23**	.21**	−.04	−.08	−.04
3. Mother’s status values			-	.03	.40**	.79**	−.05	.16*	.30*	.32**
4. Father’s moral values				-	.48**	−.03	.36**	−.15	−.23**	−.12
5. Father’s competence values					-	.46**	−.02	.18*	.07	.09
6. Father’s status values						-	−.18*	.18*	.33*	.35**
7. Mastery climate							-	−.42**	−.36**	−.14
8. Performance climate								-	.25**	.26**
9. Antisocial behavior towards teammates									-	.59**
10. Antisocial behavior towards opponents										-

*p < .05. **p < .01.

### Predicting Antisocial Behaviors: Adolescents’ Perceptions of Parents’ Sport Values and Perceived Motivational Climate

Table 3 shows both the MR models and the RWA results.

**Table 3:** The Importance of Perceived Parental Sport Values and Motivational Climate in Predicting Adolescents’ Antisocial Behaviors

Value	Multiple Regression		Relative Weight Analysis	
	β	*p*	Raw importance estimates	Rescaled estimates^a^
Adolescents’ antisocial behavior towards teammates				
Mother’s moral values	−.08	.274	.042	14.1%
Mother’s competence values	−.18	.057	.022	7.4%
Mother’s status values	.23	.004	.059	19.8%
Father’s moral values	−.07	.326	.023	7.7%
Father’s competence values	.21	.027	.019	6.4%
Father’s status values	−.07	.357	.034	11.4%
Mastery climate	−.35	.001	.074	24.8%
Performance climate	−.05	.477	.025	8.4%
Total			*R*^2^ = .298	100%
Adolescents’ antisocial behavior towards opponents				
Mother’s moral values	.03	.753	.010	4.7%
Mother’s competence values	−.26	.023	.018	8.5%
Mother’s status values	.25	.007	.066	31.3%
Father’s moral values	−.08	.343	.007	3.3%
Father’s competence values	.17	.124	.013	6.2%
Father’s status values	−.01	.866	.048	22.7%
Mastery climate	.02	.882	.005	2.4%
Performance climate	.21	.023	.044	20.9%
Total			*R*^2^ = .211	100%

^a^Rescaled estimates (%) were computed by dividing the relative weights by the total *R^2^* and multiplying by 100.

As previously mentioned, we conducted a preliminary MR separately for antisocial behaviors towards teammates and opponents, with parental sport values and perceived team motivational climate as predictors and antisocial behaviors as criterion variables. Overall, the eight predictors yielded a *R^2^* of 0.298 for antisocial behavior towards teammates and 0.211 for antisocial behavior towards opponents. From the exploration of β, in both models, mothers’ status values were a significant predictor: the more adolescents perceived their mothers as wanting them to endorse status values, the more they adopted antisocial behaviors both towards teammates and opponents. Only with regard to adolescents’ antisocial behavior towards teammates, the same was true for fathers’ competence values, whereas mothers’ competence values discouraged antisocial behaviors towards opponents. Mastery motivational climate as perceived by adolescents appeared instead to discourage antisocial behaviors towards teammates, whereas antisocial behaviors towards opponents were encouraged by a performance motivational climate.

Comparing the MR results with those of RWA, it is possible to confirm the importance of mothers’ status values, which explained 19.8% of the predicted variance of antisocial behavior towards teammates and 31.3% of the predicted variance of antisocial behavior towards opponents, and of mastery and performance climate, which explained 24.8% of the predicted variance of antisocial behavior towards teammates and 20.9% of antisocial behavior towards opponents respectively. RWA also pointed out the role of fathers’ status values in predicting antisocial behaviors towards opponents, which contributed to explain 11.4% of the predicted variance of antisocial behavior towards teammates and 22.7% towards opponents.

## Discussion and Conclusions

Research in the field of sport morality is recognizing not only the role of athletes’ personal values (e.g., Danioni & Barni, 2017; Lee et al., 2008; Lucidi et al., 2017; Šukys & Jansonienė, 2012), but is also focusing on significant adults’ (parents and coaches) values and goals in children’s sport experience (e.g., Danioni et al., 2017; Goggins, 2015; Kremer-Sadlik & Kim, 2007; Rutten et al., 2011). This study aimed at understanding the influence of adolescent athletes’ perceptions of parental sport socialization values (namely, the moral, competence and status values parents would like their children to endorse in relation to the sport activity) and of the motivational climate promoted within the team (which is deemed to be mastery or performance oriented) in shaping their antisocial behaviors towards teammates and opponents.

The findings of the current study showed that adolescents perceived their parents—both fathers and mothers—as giving the greatest importance to moral (e.g., contract maintenance and obedience) and competence values (e.g., achievement and showing skill) compared to status values (e.g., leadership and winning); this is similar to the results dealing with adolescents’ personal sport values (e.g., Danioni & Barni, 2017; Lee et al., 2008) and with children’s perceptions of their parents’ sport values (Goggins, 2015). That is, in the sport context, young athletes perceive status values as important neither for themselves nor for their parents, both in terms of parents’ personal values and, as the present study suggested, even in terms of parents’ expectations for their children.

With regard instead to the perceptions of the motivational climate promoted by the coach, our study showed that adolescents perceived their team as characterized mainly by a mastery-oriented climate, where the emphasis of the context is on participation, individual progress and task mastery, rather than by a performance-oriented climate, where the emphasis is instead on normative success and outperforming others. In line with the literature on this topic (e.g., Bortoli et al., 2012; Reinboth & Duda, 2006), since a performance-oriented climate is associated with dropout (e.g., Boiché & Sarrazin, 2009), it is likely that the adolescents who keep playing in their team sport are the ones who mostly perceive a positive environment. Moreover, it is also likely that parents who enhance moral values in the sport activity their children practice may push them to take part to sport environments which are perceived as more positive, such as the ones characterized by a mastery-oriented climate. With regard instead to the adoption of morally relevant behaviors, antisocial behaviors appeared to be quite infrequent among our participants.

Parents’ socialization sport values and the prominent motivational climate within the team turned out to be significant predictors of adolescent athletes’ antisocial behaviors towards both teammates and opponents, even though there were different patterns depending on the recipient of the behavior and, in the case of values, on the parent. As we hypothesized (H2), MR results indicated that the more adolescents perceived their mothers as wanting them to endorse status values, the higher was their tendency to adopt antisocial behaviors towards both teammates and opponents. This means that the more adolescents perceived their mothers as encouraging, for example, their winning, the more they justify the adoption of antisocial behavior in sport, probably as a means to reach the goal. Surprisingly, and in contrast with our first hypothesis (H1), perceived parents’ moral values were not involved in predicting antisocial behavior. That is, even if adolescents perceived their parents as wanting them to endorse moral values, this influence is not strong enough to discourage antisocial behaviors, as it is instead the one of the adolescents’ personal values (e.g., Danioni & Barni, 2017). We might also speculate that the reasons why adolescents adopt antisocial behaviors can explain this result; adolescents may perceive antisocial behaviors in sport as a way to affirm themselves and their supremacy over the opponents, therefore they perceive them as more related to the self-enhancement domain, rather than being related to morality.

Fathers’ competence values were instead found to be a positive significant predictor of antisocial behaviors towards teammates, whereas whenever adolescents perceived their mother as wanting them to endorse these values, not only did the recipient of the behaviors change, thus becoming the opponent, but also the direction changed, with these values discouraging antisocial behavior towards opponents. It is very important to note that the inconsistent contribution of competence values, which showed different directions when was encouraged by the mother rather than the father, was extremely reduced by using the RWA.

More in general, parents (fathers and mothers) seem to play a different role in influencing their children antisocial behaviors in sports. In considering the possible reasons of this results, we could speculate that children perceive their parents as being differently motivated in their sport value transmission, as they are in value transmission in general (Barni, Donato, Rosnati, & Danioni, 2017) and this may cause, as a consequence, a difference in the way these values then influence adolescents’ behaviors.

Finally, MR also supported our last two hypotheses (H3 and H4) about team motivational climate, although showing different patterns of influence depending on whether the recipient was a teammate or an opponent: the more adolescents perceived their team as characterized by a mastery oriented motivational climate, the less they engaged in antisocial behaviors towards teammates. Differently, the more adolescents perceived the promotion of a performance-oriented climate within the team, the more they adopted antisocial behaviors towards the opponents, thus partially disconfirming our hypothesis (H6). Despite that team motivational climate in general is more likely to affect within-team variables—as it is in our case with the relation between mastery climate and antisocial behavior towards teammates—it is likely that performance motivational climate is more strongly related to antisocial behavior in adolescent athletes than it is in adult players (Boardley & Kavussanu, 2009), thus explaining the relations we found between performance climate and antisocial behavior towards opponents. When adolescent athletes perceive the context as highly focused on achievement, this may strengthen the possibility of adopting antisocial behavior towards the ones that may be the obstacle to reach the goal, namely opponents.

RWA results were consistent with those of MR, confirming the importance of mothers’ status values in predicting antisocial behavior towards teammates and opponents, and the mastery and performance motivational climates as predictors respectively of antisocial behaviors towards teammates and opponents. More interestingly, RWA reevaluated the role of fathers’ status values in predicting antisocial behavior towards opponents, thus strengthening our second hypothesis (H2) and confirming the relevant role played by parental values influencing this specific kind of behavior (H5). Likely due to the high correlation between mothers’ and fathers’ socialization status values (see Table 2), the MR did not show this result.

Our findings suggest that both parents and coaches play a significant role in influencing the young athletes’ morally relevant behavior, specifically their voluntarily adopted antisocial behaviors both towards teammates and opponents. However, their effect appears to be quite different and therefore needs to be specifically considered, this in line with the literature that considers both their influences, for example, in the field of athletes’ motivation (Keegan, Harwood, Spray, & Lavallee, 2009) or talent development (Wolfenden & Holt, 2005).

Generally speaking, parents’ sport socialization values seem to work more as “risk factors” in enhancing antisocial behaviors, such as status values predicting antisocial behaviors, rather than “protective factors” discouraging them, since moral values were not found to reduce the frequency of their adoption. The only evident protective factor towards antisocial behaviors, and only with regard to teammates, was the mastery oriented motivational climate promoted by the coach, whereas the performance-oriented climate tended to encourage antisocial behavior towards opponents. Thus, it would be important for youth sport teams to enhance this protective factor and find a way to make it “work” even when opponents are the recipients of the behavior.

In considering simultaneously these two important social agents in adolescence, parents and coaches, our results highlighted how they both play a key role in children’s moral behavior. In particular, parental influence in terms of socialization values clearly emerged here. This result is partly in contrast with the still scarce literature that has considered both agents, since the coach usually tends to emerge as the most important source in influencing young players (Carr, Weigand, & Hussey, 1999; Guivernau & Duda, 2002; Lee & Balchin, 1996). It is, moreover, consistent in our findings that even if adolescents do not differ in the frequency of the adoption of antisocial behaviors towards a teammate or an opponent, these two patterns of the same behavior differ in their predictors.

This study includes some strong points. First, to our knowledge, it is the first study to focus on parents’ sport socialization values as perceived by their children, and it is clear from our results that this neglected topic deserves wider attention. Second, the study connected sport and family that, together with the school, are fundamental agents of socialization in daily interaction for adolescents (Prunelli, 2011). Indeed, it is essential to take into account the complex system of social relationships occurring in adolescents’ lives to fully understand the socialization process (Vandell, 2000). Third, we supplemented regression analysis with RWA, a relatively new data analysis strategy. As our predictors consisted of both parents’ socialization sport values and of the two patterns of motivational climate, which were highly correlated, this strategy allowed us to reevaluate the importance of some predictors, like the role of fathers’ status values, and to scale down the importance of others, such as parental competence values.

However, some limitations must be kept in mind when considering the results of this study. First, its cross-sectional design limited both causal inferences from the data and considerations regarding the bidirectionality of the associations found. Thus, a longitudinal development of the present research perspective would be very informative. Second, the sample was one of convenience, as participants were selected according to the willingness of their sport team to take part in the study, and the study was carried out in a single country, namely Italy. Third, adolescents were our only informants; it would be in our opinion extremely interesting as a future research direction to analyze the relation between actual parental socialization sport values—and not just children’s perceptions—with young athletes’ morally relevant behavior. Fourth, test-retest reliability of the adapted version of the YSVQ-2 was here not considered, and the consistency of the scale over time is considered as a major psychometric issue.

Moreover, future studies should also take into consideration, for example, athletes’ gender since this plays a role also in terms of value transmission process (Barni, 2009) and the kind of sport practiced, since this is likely to influence and foster the adoption of specific behaviors. Also age differences (pre-adolescents vs. late adolescents) could merit attention when considering the variables under investigation.

This study illustrates the importance of considering different factors in explaining young athletes’ morally relevant behavior in sport. It seems to us fundamental for parents and coaches to become aware of the consequences of the context they enhance and the goals and values they promote to their adolescent children and athletes. Because the relationship between parental socialization sport values and the motivational climate promoted by the coaches and the morally relevant behaviors appears evident and quite complex, significant adults such as parents and coaches need to recognize it in order to enhance children’s positive moral development.
